# Deep learning model for differentiating nasal cavity masses based on nasal endoscopy images

**DOI:** 10.1186/s12911-024-02517-z

**Published:** 2024-05-29

**Authors:** Junhu Tai, Munsoo Han, Bo Yoon Choi, Sung Hoon Kang, Hyeongeun Kim, Jiwon Kwak, Dabin Lee, Tae Hoon Lee, Yongwon Cho, Tae Hoon Kim

**Affiliations:** 1 Department of Otorhinolaryngology-Head & Neck Surgery, College of Medicine, Korea University, Seoul, Republic of Korea; 2 Mucosal Immunology Institute, College of Medicine, Korea University, Seoul, Republic of Korea; 3 Department of Radiology and AI center, College of Medicine, Korea University, Seoul, Republic of Korea; 4 Department of Computer Science and Engineering, Soonchunhyang University, Cheonan-Asan, Republic of Korea

**Keywords:** Deep learning, Artificial intelligence, Nasal endoscopy, Nasal polyps, Inverted papilloma

## Abstract

**Background:**

Nasal polyps and inverted papillomas often look similar. Clinically, it is difficult to distinguish the masses by endoscopic examination. Therefore, in this study, we aimed to develop a deep learning algorithm for computer-aided diagnosis of nasal endoscopic images, which may provide a more accurate clinical diagnosis before pathologic confirmation of the nasal masses.

**Methods:**

By performing deep learning of nasal endoscope images, we evaluated our computer-aided diagnosis system’s assessment ability for nasal polyps and inverted papilloma and the feasibility of their clinical application. We used curriculum learning pre-trained with patches of nasal endoscopic images and full-sized images. The proposed model’s performance for classifying nasal polyps, inverted papilloma, and normal tissue was analyzed using five-fold cross-validation.

**Results:**

The normal scores for our best-performing network were 0.9520 for recall, 0.7900 for precision, 0.8648 for F1-score, 0.97 for the area under the curve, and 0.8273 for accuracy. For nasal polyps, the best performance was 0.8162, 0.8496, 0.8409, 0.89, and 0.8273, respectively, for recall, precision, F1-score, area under the curve, and accuracy. Finally, for inverted papilloma, the best performance was obtained for recall, precision, F1-score, area under the curve, and accuracy values of 0.5172, 0.8125, 0.6122, 0.83, and 0.8273, respectively.

**Conclusion:**

Although there were some misclassifications, the results of gradient-weighted class activation mapping were generally consistent with the areas under the curve determined by otolaryngologists. These results suggest that the convolutional neural network is highly reliable in resolving lesion locations in nasal endoscopic images.

## Background

Nasal polyps (NPs) are inflammatory products of the nasal sinus tissue, which are usually bilateral, and benign [1]. However, if a nasal mass grows on only one side of the nasal cavity, the possibility of a potential tumor should be considered; the most common type of which is inverted papilloma (IP) [2]. IPs are related to the human papilloma virus, prone to recur after being surgically removed, and occasionally transform into a malignant tumor [3]. The percentage of malignant transformation to squamous cell carcinoma is 5–13% and the five-year survival rate after malignant transformation is less than 50% [4]. Because prognosis of IPs is worse than that of NPs, the preoperative differential diagnosis of these two diseases is of great importance for selecting the appropriate surgical methods and the prognosis of patients [5]. However, NPs and IPs often have similar appearance, and it is difficult to distinguish them in clinical settings [6].

Endoscopy is commonly used for clinical diagnosis of nasal masses [7]. The final diagnosis of nasal masses must be combined with computed tomography (CT) and pathologic findings [8]. However, nasal endoscopy is a more rapid and non-invasive method in outpatient examination, although unilateral nasal polyps can easily be misdiagnosed by endoscopy alone. Before the pathological results are available after surgery, the preliminary diagnosis often depends on the experience of the physician, who can easily make mistakes. A study in which several rhinologists evaluated the results of different types of nasal endoscopy found significant differences in outcome evaluation [9].

Meanwhile, recent studies indicate that machine learning algorithms, particularly convolutional neural networks, excel in visual object recognition [10] and surpass humans in object recognition [11]. Studies have demonstrated the feasibility of artificial intelligence (AI) in diagnosing various lesions and patterns in medical imaging [12, 13]. Similarly, [14] showed the potential of a deep learning-based diagnosis system for the automatic classification of NPs and IPs. Although this has been developed using deep learning algorithms based on the transfer learning strategy used in this study, there are limitations to classifying three classes. Curriculum learning [15], involving the step-by-step training of more sophisticated concepts, could partially solve these complex challenges. Using this approach, [16] proposed a curriculum for refining the analysis of complex full images by initially training on lesion-specific patch images from chest X-rays.

Therefore, using machine learning for computer-aided diagnosis of nasal endoscopic images can provide more accurate results based on curriculum learning. Deep learning of nasal endoscopic images can support the assessment of NPs and IP. Our algorithms were compared with [16] algorithms and general deep learning without curriculum learning. This study presents a computer-aided diagnosis system based on deep learning. We further demonstrate its potential for clinical applications.

## Methods

### Participants and grouping

Patients who attended a tertiary medical institution in South Korea between January 1, 2016, and May 31, 2019, and underwent septoplasty with submucosal turbinoplasty or endoscopic sinus surgery were involved in the study. Patients without a mass in the nasal cavity who underwent septoplasty were assigned to the normal group. Patients diagnosed pathologically with NPs were assigned to the NP group, while those diagnosed with IP after endoscopic sinus surgery (ESS) were assigned to the IP group. Objects with poor photo quality or those that could not be obtained due to computer errors were excluded. Endoscopic images were examined by the rhinologist (T.H.K.) using a 4 mm rigid telescope (Olympus Medical Systems Corp., Tokyo, Japan) and a HDTV endoscope video processor system (VISERA ELITE OTV-S190; Olympus Medical Systems Corp., Tokyo, Japan). Revision surgeries with recurred nasal masses were excluded from the data. The study was approved by the Institutional Review Board of the Korea University Hospital (approval number: 2019AN0264). Furthermore, we confirm that all experiments were performed in accordance with relevant guidelines and regulations. Since this was a retrospective study, informed consent was not obtained from the participants, and the National Committee for Ethics waived the informed consent for this study.

The nasal endoscopic images were collected from the Korea University Anam Hospital (KUAH). The nasal endoscopic images of normal subjects (490 cases) and patients (952 cases), including NPs (775 cases) and IP (177 cases), in the full-resolution images randomly divided into training, tuning, and testing sets in a ratio of 7:1:2, were enrolled at KUAH (Table 1). Normal subjects and patients with NPs and IPs were determined based on paranasal sinus CT and postoperative pathologic findings. As a gold standard, two otolaryngologists defined lesions based on in-house regions of interest (ROIs) and manually made annotations for the image lesions. Patch images were generated from the full-resolution images of representative learnings (Fig. 2 and Table 1). The number of patch images from normal subjects and patients, including NPs (3096 cases) and IPs (708 cases), are listed in Table 1.

**Table 1:** Number of nasal endoscopic images for training, tuning, and testing

Endoscopic Patch images	Total	KUAH (Training)	KUAH (Tuning)	KUAH (Testing)
Normal	1960	1456	168	336
Abnormal	3804	2860	288	656
NPs	3096	2324	232	540
IP	708	536	56	116
Endoscopic whole images	Total	KUAH (Training)	KUAH (Tuning)	KUAH (Testing)
Normal	490	364	42	84
Abnormal	952	715	72	165
NPs	775	581	58	136
IP	177	134	14	29

*KUAH* Korea University Anam Hospital, *NPs* Nasal polyps, *IP* inverted papilloma

### Curriculum learning strategy using training patches and full-sized images

InceptionResNetV2 [17] was configured with an inception layer and residual connections, which included various convolutional filters connected to residual blocks. These blocks not only mitigated the gradient descent issues associated with the depth of the deep learning network but also shortened the training duration. Curriculum learning to infer multiple classes generally requires different and various datasets due to the complexity of medical images. Full-resolution nasal endoscope images contain complex patterns, including lesions, organs, and tissues, which complicates training with limited datasets. The given image underwent standard image preprocessing (bi-linear interpolation) and was resized to a fixed size of 512 × 512 pixels to closely resemble a general natural image. In a previous study, a simple curriculum learning strategy [16] to train various lesion patterns in two steps showed much better performance. To address the issue of imbalance, the loss for each class was adjusted by multiplying it by its respective weight [16]. A straightforward curriculum learning strategy [16], involving two steps, was employed to train intricate disease patterns. In the first step, the pre-trained ResNet-50 network from the ILSVRC dataset was fine-tuned using lesion-specific patch images. In the second step, the network was fine-tuned using full-sized images because of the difference in distribution between patches and full-sized images.

**Fig. 1:**
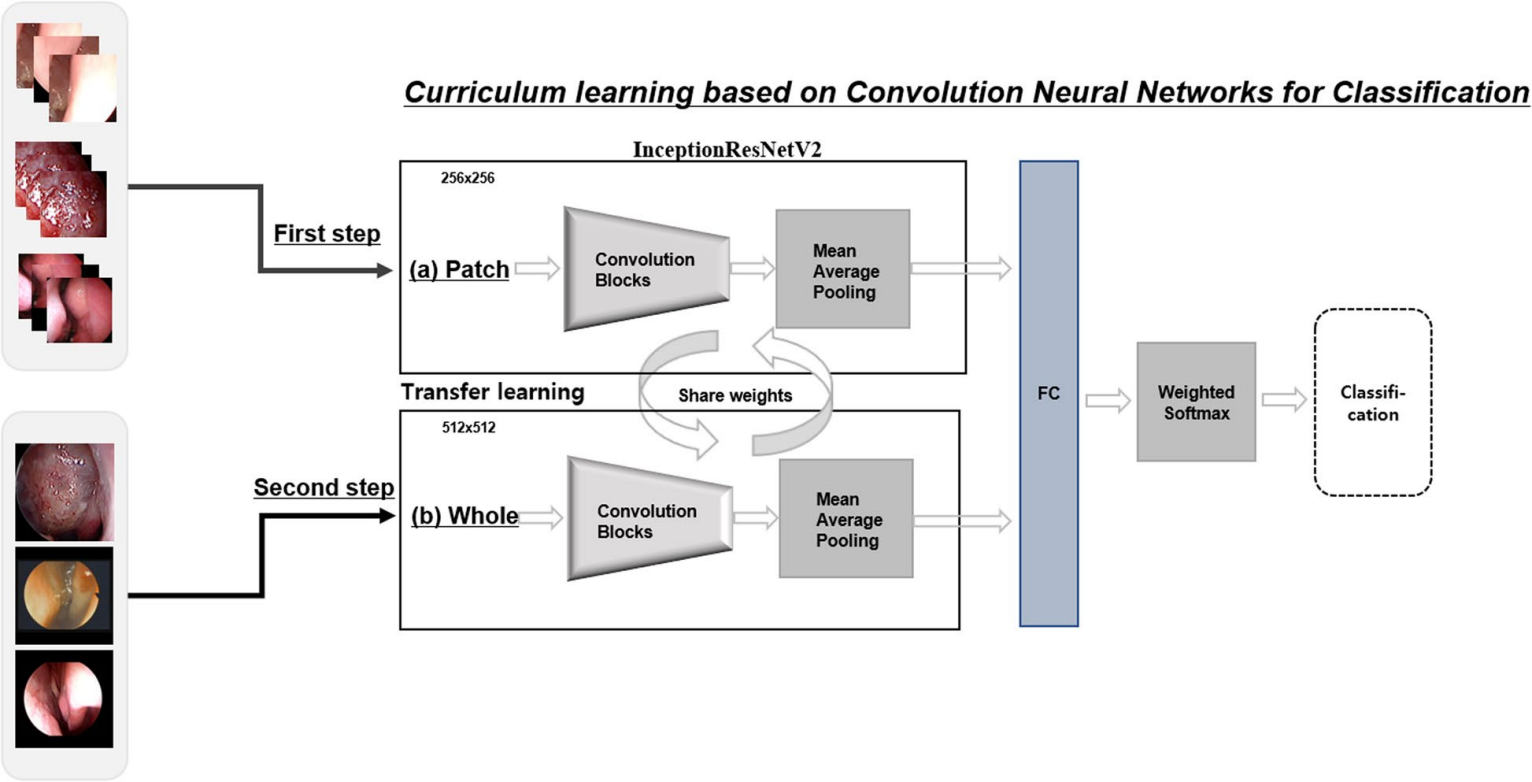
Architecture for curriculum learning: (**a**) first step based on pre-trained weights (InceptionResNetV2 with ImageNet) with patch images; (**b**) second step with full-resolution nasal endoscope images based on the weights of (**a**)

**Fig. 2:**
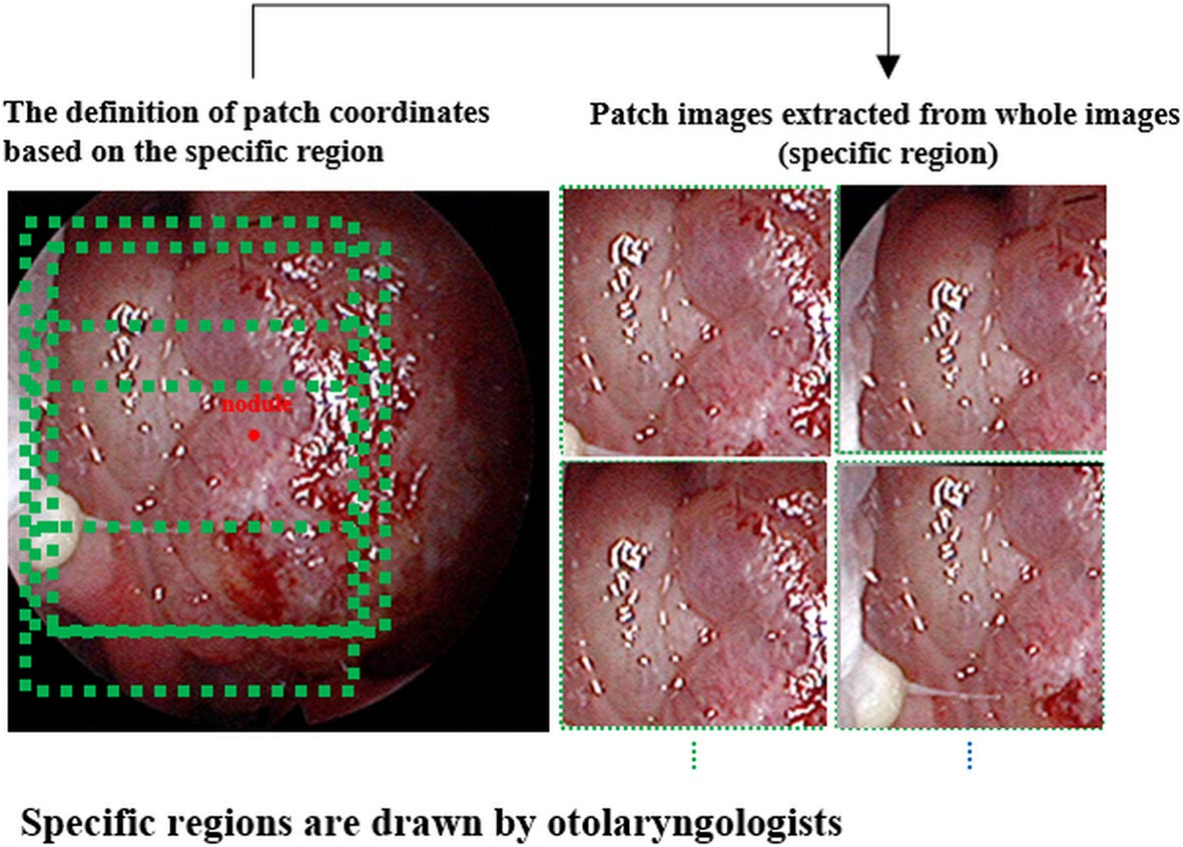
Methods for extracting patch images using the central areas of lesions for curriculum learning

We employed curriculum learning as a representative learning strategy to classify lesions and normal images from nasal endoscopies. This approach aimed to improve model performance with a small dataset and address the dataset’s imbalance across three classes, as illustrated in Fig. 1. Since our datasets consist of an unbalanced form, we used patch images extracted from the features of the lesion to obtain more diversity. In addition, to improve training and tuning, different patch datasets were extracted from areas around the points selected by expert otolaryngologists to better train the regional patterns of lesions or normal tissue, as shown in Figs. 1 and 2. The patch images (256 × 256) were reduced to half of their raw resolution to ensure they contained multiple lesions around their central region. Subsequently, the network was fine-tuned using full-resolution nasal endoscope images to compensate for discrepancies between the full-sized and patch images.

In the first step, the InceptionResNetV2network, a pre-trained model using the ImageNet Large Scale Visual Recognition Competition (ILSVRC) [18], was trained using the extracted patches of nasal endoscope images, as shown in Fig. 2. These deep convolutional neural networks (CNNs) can learn general or different medical image features using other domain datasets without training the network from scratch. In the second step, we fine-tuned only the last layer of the model trained in the first step using all images. In addition, geometric enhancements such as augmentation using zoom, rotation, and shifting were used for training and tuning. Due to potential variations in patterns among manufacturers in these images, we conducted a random application of sharpening and blurring techniques during the training and tuning phases. This approach aimed to enhance the performance of the model with diverse image variations. Furthermore, our training data incorporated additional augmentation techniques, including rotation (±10°), zoom (±10%), and shifting (±10%). These enhancements were performed to improve the model’s resilience to variations not related to radiological classes using Python 3.6.

All datasets for training, tuning, and testing were loaded onto a GPU platform running Ubuntu 20.04, NVIDIA Toolkit 460.80, three 24 GB Titan RTXs and one 48GB Quadro RTX 800 graphics cards, and cuDNN 11.2 (NVIDIA Corporation) with Keras running on top of TensorFlow. We used hyperparameters in various training settings. To mitigate tuning errors in selecting optimized models, the backpropagation algorithm was executed over 25 training epochs using a batch size of eight. We used the ADAM optimizer and weighted loss with an initial learning rate of 0.001 for three-way classification. The cross-entropy cost function in binary classification (1.1) is expressed as follows:

1.1 $$\textrm{Loss}\ \left(y,f\right)=-y\ \log\ f-\left(1-y\right)\ \log \left(1-f\right)$$

where *f* and *y* denote the inferred probability and the corresponding desired output, respectively.

### Statistical analysis

We evaluated diagnostic performance for inference of NPs, IP, and normal using five-fold cross-validation with terms forming the confusion matrix, as follows: True positive (TP) is the number of labels correctly classified as positive by the algorithms; true negative (TN) is the number of labels correctly classified as negative by the algorithms; false positive (FP) is the number of labels incorrectly classified as positive by the algorithms; and false negative (FN) is the number of labels incorrectly classified as negative by the algorithms. Finally, the performance of multiple classifications based on the full-resolution images of the nasal endoscope was evaluated using four methods: recall, precision, F1-score, and accuracy with the scikit-learn Python library, as follows:

1.2 $$\textrm{Recall}=\frac{TP\ }{TP+ FN}$$

1.3 $$\textrm{Precision}=\frac{TP\ }{TP+ FP}$$

1.4 $$\textrm{F}1-\textrm{score}=\frac{2\left( Precision\ x\ Recall\right)\ }{Precision\ x\ Recall}$$

1.5 $$\textrm{Accuracy}=\frac{TN+ TP\ }{TN+ TP+ FN+ FP}$$

Accuracy is the ratio between the number of correctly classified test samples and the total number of test samples.

For the multiclass case, the area under the curve (AUC) was analyzed using the receiver operating characteristic (ROC) (1.17.0.1) within the R package. Our model was also compared with another model [17] using paired t-tests for statistical significance, which was set at *P* < 0.05.

## Results

### Comparison between our and another algorithm

To predict NPs, IP, and normal in nasal endoscope images, we used a curriculum learning-based deep learning network as the backbone, InceptionResNetV2, shown in Fig. 1, which was trained and tuned with five-fold cross-validation. The KUAH dataset (normal, 84 cases; NPs, 136 cases; and IP, 29 cases) was used for testing. This training model extracted probabilities per image to be classified as either normal or other lesions corresponding to other classes. In statistical analysis, binary labeling was used to evaluate recall, precision, F1-score, and accuracy. With three classes, binary labeling was created by combining one class with the other two, and a total of three sets of statistical metrics were calculated based on these binary labels.

**Fig. 3:**
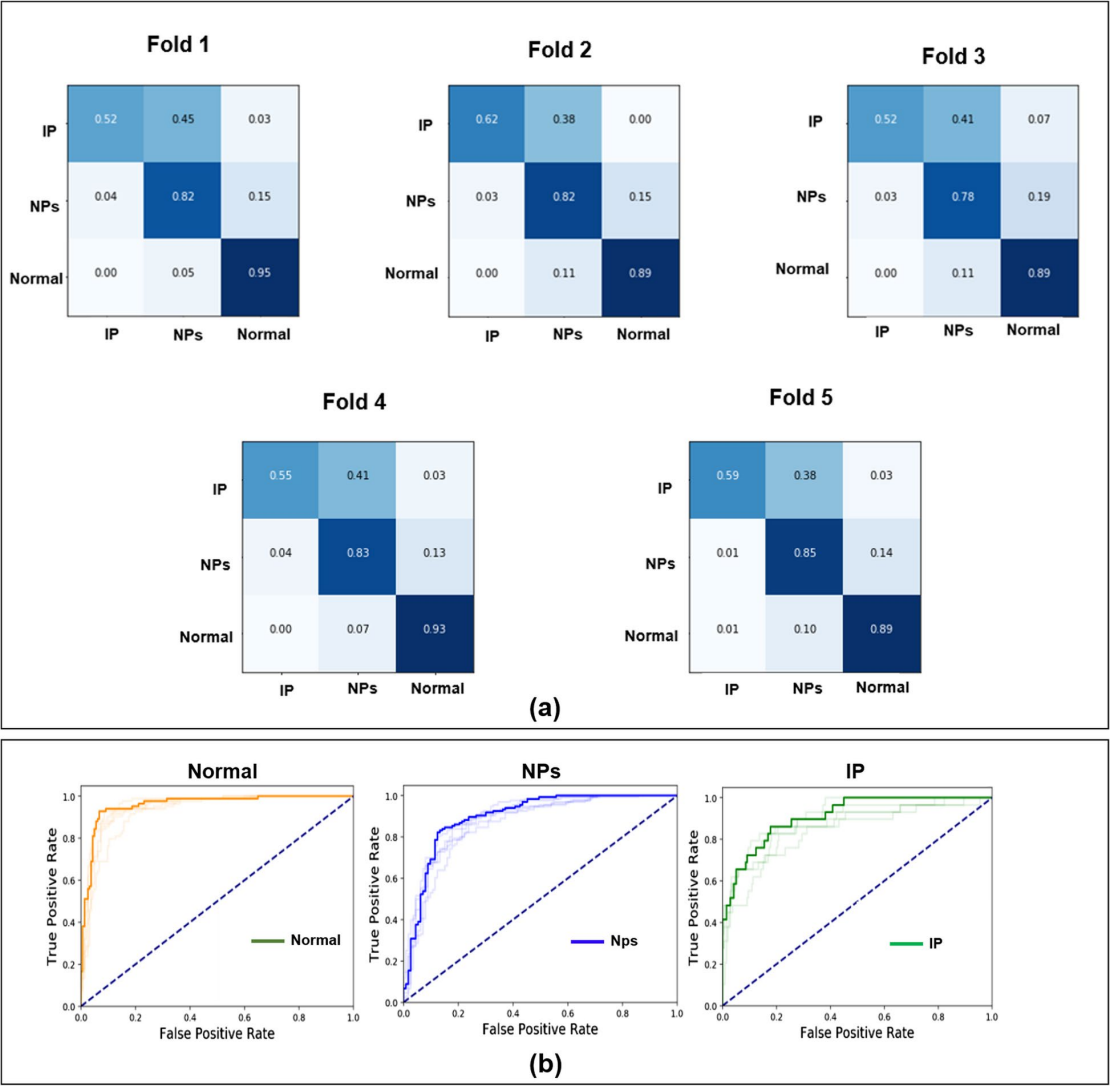
Results of classification for normal, NPs, and IP: (**a**) confusion matrix on test datasets (KUAH) and (**b**) mean receiver operating characteristic (ROC) curve for five-folds. Note: Korea University Anam Hospital (KUAH); nasal polyps (NPs); inverted papilloma (IP). *p*-values > 0.05 (fold2:0.91; fold3:0.83; fold4:0.14; fold5:0.14) for the best performance among five-folds and other folds and *p* value < 0.05 (IP: 2.2e-16; NPs: 1.64e-06; Normal: 0.004) for the curriculum learning backbone network: InceptionResNetV2 and CNN

Each result of the five-fold cross-validation is shown in Fig. 3 and Table 2. The following averages were obtained: 0.90 ± 0.04 for recall, 0.78 ± 0.03 for precision, 0.84 ± 0.03 for F1-score, 0.95 ± 0.02 for AUC, and 0.82 ± 0.02 for accuracy. The best performance was observed for the first fold. The normal scores for the network with the best performance were 0.90 ± 0.04 for recall, 0.78 ± 0.03 for precision, 0.84 ± 0.03 for F1-score, 0.95 ± 0.02 for AUC, and 0.82 ± 0.02 for accuracy, respectively. The NP scores for the network with the best performance were 0.82 ± 0.02 for recall, 0.85 ± 0.01 for precision, 0.84 ± 0.02 for F1-score, 0.88 ± 0.01 for AUC, and 0.82 ± 0.02 for accuracy, respectively. The IP scores for the network with the best performance were 0.56 ± 0.04, 0.81 ± 0.03, 0.66 ± 0.04, 0.87 ± 0.03, and 0.82 ± 0.02 for recall, precision, F1-score, AUC, and accuracy, respectively.

**Table 2:** Classification results (Normal, NPs, and IP) on the KUAH

	Class	Recall	Precision	F1-score	AUC for multiclass	Total Accuracy
Curriculum learning backbone network: InceptionResNetV2	Normal	0.90 ± 0.04	0.78 ± 0.03	0.84 ± 0.03	0.95 ± 0.02	0.82 ± 0.02
	NPs	0.82 ± 0.02	0.85 ± 0.01	0.84 ± 0.02	0.88 ± 0.01	
	IP	0.56 ± 0.04	0.81 ± 0.03	0.66 ± 0.04	0.87 ± 0.03	
InceptionResNetV2 without curriculum learning	Normal	0.87 ± 0.02	0.78 ± 0.06	0.82 ± 0.04	0.93 ± 0.03	0.80 ± 0.03
	NPs	0.86 ± 0.04	0.80 ± 0.02	0.83 ± 0.05	0.85 ± 0.01	
	IP	0.28 ± 0.04	0.99 ± 0.03	0.43 ± 0.03	0.89 ± 0.03	
[17] CNN-ResNet152	Normal	0.79 ± 0.02	0.78 ± 0.05	0.82 ± 0.03	0.93 ± 0.01	0.79 ± 0.02
	NPs	0.80 ± 0.03	0.81 ± 0.03	0.81 ± 0.02	0.88 ± 0.02	
	IP	0.50 ± 0.09	0.68 ± 0.07	0.57 ± 0.08	0.84 ± 0.03	

*KUAH* Korea University Anam Hospital, *NPs* Nasal polyps, *IP* inverted papilloma

Our model was compared with another model without curriculum learning (*p* < 0.05), and the corresponding values of normal for those were as follows: 0.87 ± 0.02 for recall, 0.78 ± 0.06 for precision, 0.82 ± 0.04 for F1-score, 0.93 ± 0.31 for AUC, and 0.80 ± 0.03 for accuracy, and those of NPs and IP were as follows: 0.86 ± 0.04 and 0.28 ± 0.04 for recall, 0.80 ± 0.02 and 0.99 ± 0.03 for precision, 0.83 ± 0.05 and 0.43 ± 0.03 for F1-score, 0.85 ± 0.01 and 0.89 ± 0.03 for AUC, and 0.80 ± 0.03 for accuracy, respectively (Table 2).

Our model was compared with another model (p < 0.05), and the corresponding values of normal for the other model [17] were as follows: 0.79 ± 0.02 for recall, 0.78 ± 0.05 for precision, 0.82 ± 0.03 for F1-score, 0.93 ± 0.01 for AUC, and 0.79 ± 0.02 for accuracy, and those of NPs and IP were as follows: 0.80 ± 0.03 and 0.50 ± 0.09 for recall, 0.81 ± 0.03 and 0.68 ± 0.07 for precision, 0.81 ± 0.02 and 0.57 ± 0.08 for F1-score, 0.88 ± 0.03 and 0.84 ± 0.03 for AUC, and 0.79 ± 0.02 for accuracy, respectively in Table 2.

### Comparison between our algorithms and clinicians’ analyses

**Fig. 4:**
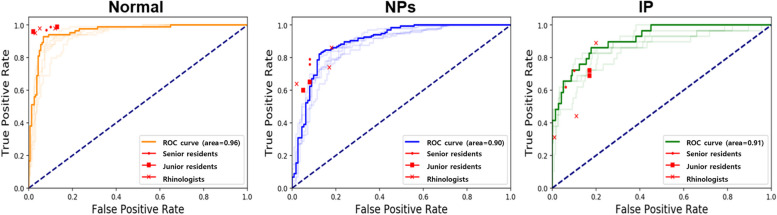
Visual scoring of classification for normal, NPs, and IP: (**a**) normal, (**b**) NPs, and (**c**) IP on test datasets (KUAH) between deep learning and otolaryngologists

The visual scoring of clinicians was analyzed by seven human experts, and this analysis was compared with the performance of our deep learning model. Seven otolaryngologists analyzed the images with the test dataset (249 images), including normal and abnormal images (NPs and IP), as shown in Table 1. Three of the seven were board-certified experts in nasal endoscopy; two were senior residents with clinical experience of over 3 years, and the remaining two were junior residents with a maximum clinical experience of 2 years. We compared our deep-learning algorithm against human performance by analyzing the confusion matrices for three classifications. Additionally, we assessed the AUC to evaluate the performance of each class, comparing the deep learning model with seven experts (see Fig. 4).

Table 3 displays the classification performance results for both our model and the experts. First, the corresponding values of normal for our algorithm are as follows: 0.90 ± 0.04 for recall, 0.78 ± 0.03 for precision, 0.84 ± 0.03 for F1-score, 0.95 ± 0.02 AUC, and 0.82 ± 0.02 for accuracy. Those for the human experts were as follows: 0.98 ± 0.01 for recall, 0.85 ± 0.02 for precision, 0.91 ± 0.01 for F1-score, 0.95 ± 0.02 for AUC, and 0.93 ± 0.01 for accuracy.

**Table 3:** Comparison of the classification (normal, NPs, and IP) performance in the KUAH dataset between deep learning and humans

	Class	Recall	Precision	F1-score	AUC for multiclass	Accuracy for each class
Curriculum learning backbone network: InceptionResNetV2	Normal	0.90 ± 0.04	0.78 ± 0.03	0.84 ± 0.03	0.95 ± 0.02	0.82 ± 0.02
	NPs	0.82 ± 0.02	0.85 ± 0.01	0.84 ± 0.02	0.88 ± 0.01	0.80 ± 0.02
	IP	0.56 ± 0.04	0.81 ± 0.03	0.66 ± 0.04	0.87 ± 0.03	0.85 ± 0.02
Seven otolaryngologists	Normal	0.98 ± 0.01	0.85 ± 0.02	0.91 ± 0.01	0.95 ± 0.02	0.93 ± 0.01
	NPs	0.71 ± 0.08	0.91 ± 0.02	0.80 ± 0.05	0.81 ± 0.03	0.80 ± 0.04
	IP	0.64 ± 0.08	0.48 ± 0.10	0.51 ± 0.01	0.76 ± 0.04	0.86 ± 0.04
Rhinologists	Normal	0.98 ± 0.02	0.87 ± 0.05	0.92 ± 0.03	0.95 ± 0.02	0.94 ± 0.02
	NPs	0.75 ± 0.11	0.89 ± 0.08	0.81 ± 0.04	0.81 ± 0.02	0.81 ± 0.03
	IP	0.55 ± 0.31	0.54 ± 0.31	0.46 ± 0.07	0.72 ± 0.11	0.86 ± 0.05
Senior residents	Normal	0.98 ± 0.02	0.85 ± 0.01	0.91 ± 0.01	0.95 ± 0.02	0.94 ± 0.01
	NPs	0.78 ± 0.03	0.92 ± 0.01	0.84 0.01	0.85± 0.11	0.84 ± 0.01
	IP	0.67 ± 0.06	0.54 ± 0.07	0.60 ± 0.01	0.80 ± 0.02	0.89 ± 0.01
Junior residents	Normal	0.98 ± 0.03	0.83 ± 0.04	0.90 ± 0.02	0.94 ± 0.02	0.93 ± 0.01
	NPs	0.62 ± 0.04	0.92 ± 0.03	0.74 ± 0.02	0.78 ± 0.02	0.76 ± 0.01
	IP	0.71 ± 0.02	0.36 ± 0.02	0.47 ± 0.01	0.77 ± 0.01	0.82 ± 0.01

*KUAH* Korea University Anam Hospital, *NPs* Nasal polyps, *IP* inverted papilloma

Second, the corresponding values of NPs for our algorithm were as follows: 0.82 ± 0.02 for recall, 0.85 ± 0.01 for precision, 0.84 ± 0.02 for F1-score, 0.88 ± 0.01 for AUC, and 0.80 ± 0.02 for accuracy. Those for the human experts were as follows: 0.71 ± 0.08 for recall, 0.91 ± 0.02 for precision, 0.80 ± 0.06 for F1-score, 0.81 ± 0.03 for AUC, and 0.80 ± 0.04 for accuracy.

The corresponding values of IP for our algorithm were as follows: 0.56 ± 0.04 for recall, 0.81 ± 0.03 for precision, 0.66 ± 0.04 for F1-score, 0.87 ± 0.03 for AUC, and 0.85 ± 0.02 for accuracy. Those for the human experts were as follows: 0.64 ± 0.08 for recall, 0.48 ± 0.10 for precision, 0.51 ± 0.01 for F1-score, 0.76 ± 0.04 for AUC, and 0.86 ± 0.04 for accuracy.

Our model achieved similar AUCs to those of the seven human experts but showed lower accuracy and recall than the experts (particularly for the normal group). Although the experts outperformed our model in the normal group, our model outperformed the experts in the NP classification (recall: 0.82 ± 0.02 vs. 0.71 ± 0.08; AUC: 0.88 ± 0.01 vs. 0.81 ± 0.03; *p* < 0.05).

**Fig. 5:**
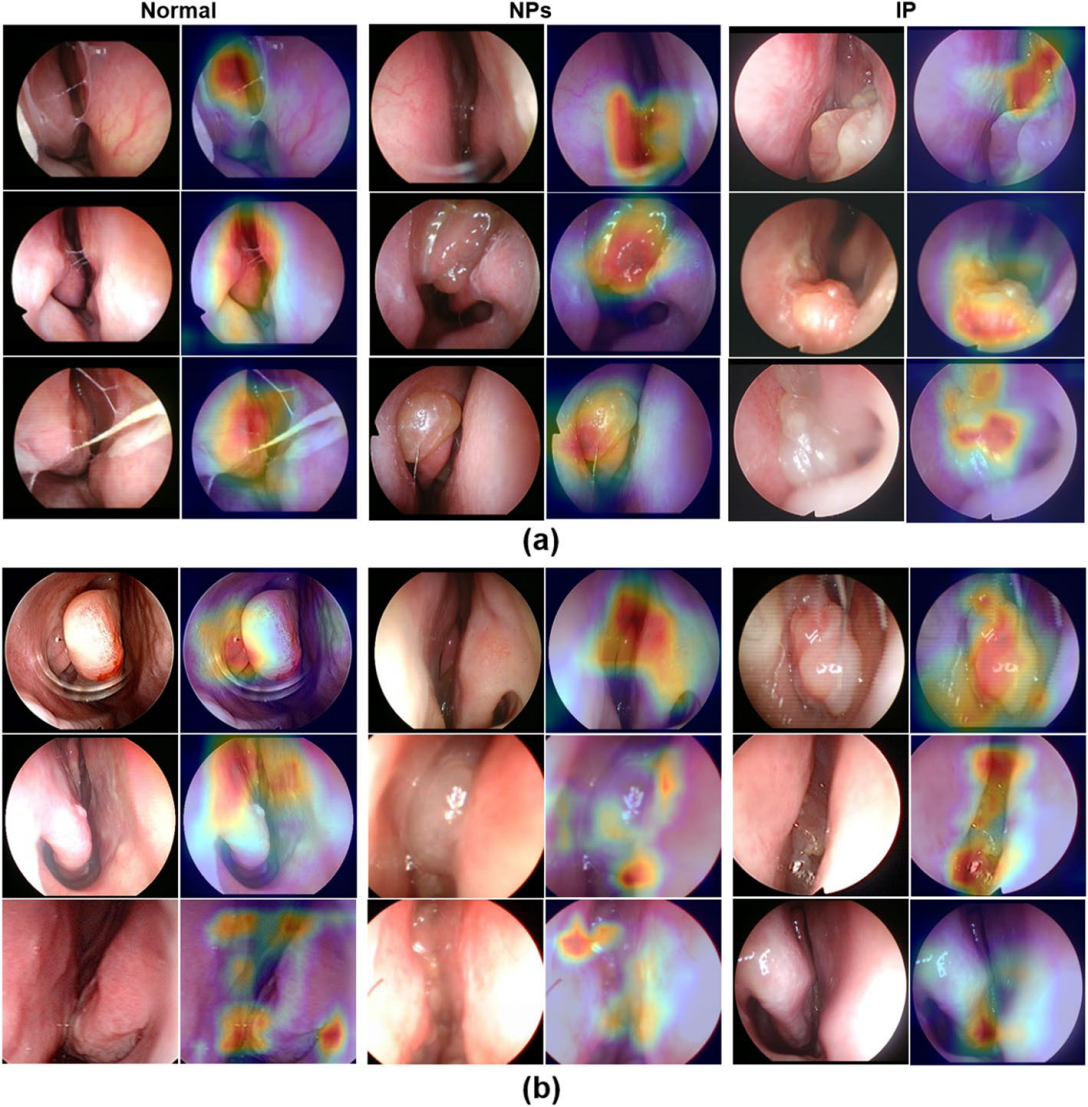
Grad-CAM results of the best model among five-folds for normal, NPs, and IP on nasal endoscope images. **a** Positive results for the classification of normal, NPs, and IP in the nasal endoscope images and the corresponding heat maps for our model. **b** Negative results for the classification of normal, NPs, and IP in the nasal endoscope images and the corresponding heatmaps for our model. Grad-CAM results for each class were extracted independently, with the Grad-CAMs for each class extracted by deep learning and being consistent with specific regions delineated by experts

For the test, the best model among the five-fold cross-validation was determined using gradient-weighted class activation mapping (Grad-CAM) [19] for normal and abnormal (NPs and IPs) images after training based on curriculum learning, as shown in Fig. 5. Although the Grad-CAM results were generally consistent with the AUC results of otolaryngologists, our deep learning model misclassified some patients with normal or other lesions (NPs and IPs) (Fig. 4). Most misclassifications made by humans were NPs among normal and other lesions.

## Discussion

In this study, we demonstrated the detection and classification of nasal masses using CNNs. We demonstrated that the trained CNNs could discriminate between NPs and IPs with high accuracy using limited learning samples. Our study evaluated the model’s utility, particularly for primary physicians without specialized training in otolaryngology. The clinical role of this deep learning model is to enable not only the primary physicians but also the otolaryngologists to obtain more accurate information from the nasal endoscopy images. This supports the concept that optimizing the deep learning architecture is useful and effective in clinical nasal endoscopic practice. Although, it is important to develop deep learning algorithms with a large volume of dataset, our algorithm demonstrated superior accuracy compared to other models in scenarios with fewer total samples [17], as detailed in Table 2.

Several articles have been published on the application of CNNs in otolaryngology endoscopy. After deep learning based on 6066 otoscopic images from 2022 participants, the CNN’s accuracy in diagnosing otitis media based on tympanic membrane images achieved 93.4%, and the diagnosis level reached the level of an associate professor of otolaryngology [20]. A CNN model based on deep learning of over 4000 laryngoscope images, including cysts, nodules, polyps, leukoplakia, and papilloma, demonstrates better diagnostic performance than clinicians, with an average AUC of 0.95 in distinguishing papilloma an F1-score of 0.870 [21]. In addition to otolaryngology endoscopy, CNNs have also been employed to conduct research on endoscopy in other fields. In gastroenterology, for example, a trained CNN model demonstrated an accuracy of 91.2% in distinguishing gastrointestinal stromal tumors and leiomyomas in endoscopic ultrasound images [22]. In colorectal polyp detection, a CNN model accurately classified 83% of polyps from images and accurately identified 97% of adenomas under white light images [23]. CNNs have also achieved 97% sensitivity and 94% accuracy in detecting and classifying nasal cytology images [24].

Curriculum learning based on the centering of lesions in the patch image is crucial for training deep learning models with limited medical datasets and for interpreting the localization and features of lesions. In the first stage, the patch image used for the pre-trained model reflects the shape and texture of different lesions in the medical dataset (endoscopic images or others). As shown in Table 2, our algorithm outperformed the other models [17] and algorithms (InceptionResNetv2) without curriculum learning in classifying normal and abnormal endoscopic images. This strategy is important for diagnosing patients and assisting clinicians in the medical setting.

In this study, we used multiple parameters to analyze the performance of our algorithm. To eliminate the deviation caused by the unbalanced composition of images in the training, tuning, and testing sets in the training stage, we verified it with five-fold cross-validations. By employing the curriculum learning strategy, only 5764 images were used, achieving an average accuracy of 0.82 ± 0.02. Furthermore, the performance is improved by reflecting the main characteristics of the lesion well. Moreover, the attention mechanism of the trained model was consistent with the local lesion-related areas, particularly with respect to those that experienced otolaryngologists focus on during nasal endoscopy. The ROC curves demonstrate that the evaluations made by otolaryngologists and our Grad-CAM were generally similar. Otolaryngologists made some incorrect assessments, most of which were NPs. Although the human experts performed better in classifying normal images, our model performed better than the experts in classifying NPs. This result may have been observed because the experts can easily distinguish an endoscopic picture of normal nasal turbinate from an abnormality, while our model could have detected the turbinate as an NP, which has a similar soft mucosal texture. Our model exhibited improved performance in classifying NPs, despite the inherent difficulty in distinguishing NPs in nasal endoscopy, even for experienced professionals. Rhinologists often resort to collecting biopsy samples from lesions to conclusively determine whether they are NPs or not. Therefore, the promising performance of our model in NP classification is noteworthy, as it surpassed the clinical impressions of experts. Therefore, it is plausible that the proposed model is not only helpful to inexperienced otolaryngologists but also to experienced physicians.

Studies have been conducted on machine learning for application in CT images [25] and pathological slides [26] that achieved good results. In our next work, we will not only include endoscopic images but also consider adding CT images and pathological slice images to observe the accuracy of the model after training in comparison with this study’s results. We will experiment to compare the model after training with the performance of human physicians using different types of images, such as endoscopic, CT, and pathological images. In addition, this model was trained only to diagnose diseases. However, we will consider whether the model can still show outstanding ability in disease prevention and disease prognosis assessment when various images of diseases at each pathogenic stage are used to train the model.

This study has several limitations. First, the number of images was relatively small, particularly the IP images, to perform conventional deep learning. To overcome the limited sample size, we used the curriculum learning strategy to train patches and full-sized images. Although our CNN model achieved high accuracy, more images will be required for further study, and we will develop advanced algorithms to classify normal, NPs, and IP. Second, since this study was conducted from a single tertiary referral hospital located in Seoul, South Korea, it was difficult to collect endoscopic images from different epidemiologic backgrounds such as race or residence to further investigate and verify the CNN model. There was a lack of endoscopic images from other hospitals to further investigate and verify the CNN model. Third, the training images used in this study were selective. Most of the training images were clear and typical. To verify CNNs, a significant number of various images are needed. Therefore, in future studies, we plan to collect various images, develop more powerful CNNs to better fit the actual clinical environment, and analyze experiment results using various ablation studies, such as curriculum learning based on other models [17]. Finally, the proposed CNN model could distinguish only three types of images. When the model encounters images of unseen diseases, it may make an incorrect diagnosis due to its limitations. This requires a significant number of nasal endoscopic images of other diseases to be further investigated. In the future, a multi-center study with larger data validated by a larger group of clinicians can be conducted to produce more rigorous results.

## Conclusion

This study revealed potential results, indicating that the proposed deep learning algorithm effectively detects nasal masses in endoscopic images of the nasal cavity. It provides a reference for clinicians and can help inexperienced examiners distinguish nasal endoscopy images. However, further image accumulation and prospective studies are required to further improve its reliability and accuracy.

## Data Availability

The datasets used in the manuscript are available from the corresponding author upon reasonable request.

## Abbreviations

NPs: Nasal polyps
IP: Inverted papilloma
CT: Computed tomography
AI: Artificial intelligence
ESS: Endoscopic sinus surgery
KUAH: Korea University Anam Hospital
ROIs: Regions of interest
ILSVRC: ImageNet Large Scale Visual Recognition Competition
CNNs: Convolutional Neural Networks
TP: True positive
TN: True negative
FP: False positive
FN: False negative
AUC: Area under the curve
ROC: Receiver operating characteristic
Grad-CAM: Gradient-weighted class activation mapping
