# Correction: Deep learning model for differentiating nasal cavity masses based on nasal endoscopy images

**DOI:** 10.1186/s12911-024-02562-8

**Published:** 2024-06-03

**Authors:** Junhu Tai, Munsoo Han, Bo Yoon Choi, Sung Hoon Kang, Hyeongeun Kim, Jiwon Kwak, Dabin Lee, Tae Hoon Lee, Yongwon Cho, Tae Hoon Kim

**Affiliations:** 1 Department of Otorhinolaryngology-Head & Neck Surgery, College of Medicine, Korea University, Seoul, Republic of Korea; 2 Mucosal Immunology Institute, College of Medicine, Korea University, Seoul, Republic of Korea; 3 Department of Radiology and AI center, College of Medicine, Korea University, Seoul, Republic of Korea; 4 Department of Computer Science and Engineering, Soonchunhyang University, Cheonan-Asan, Republic of Korea

**Correction to: Tai et al. BMC Medical Informatics and Decision Making (2024) 24:145**

10.1186/s12911-024-02517-z

Following the publication of the Original Article, the author reported an incorrect Funding from the ICT Creative Consilience program as follows: IITP-2023-2020-0-01819.

The correct number is IITP-2024-2020-0-01819.

The Original Article has been corrected.

